# Genome-Wide Identification and Transcriptional Expression of the *PAL* Gene Family in Common Walnut (*Juglans Regia* L.)

**DOI:** 10.3390/genes10010046

**Published:** 2019-01-15

**Authors:** Feng Yan, Huaizhu Li, Peng Zhao

**Affiliations:** 1 Key Laboratory of Resource Biology and Biotechnology in Western China, Ministry of Education, College of Life Sciences, Northwest University, Xi’an, Shaanxi 710069, China; fengyan115@163.com; 2 School of Chemistry and Chemical Engineering, Xianyang Normal University, Xianyang 712000, China; lihuaizhu121@126.com

**Keywords:** common walnut, phenylalanine ammonia-lyase (PAL), evolution, phylogenetic, expression profiling, female and male flowers

## Abstract

*Juglans regia* L. is an economically important crop cultivated worldwide for its high quality and quantity of wood and nuts. Phenylalanine ammonia-lyase (PAL) is the first enzyme in the phenylpropanoid pathway that plays a critical role in plant growth, development, and adaptation, but there have been few reports of the *PAL* gene family in common walnut. Here, we report a genome-wide study of *J. regia* *PAL* genes and analyze their phylogeny, duplication, microRNA, and transcriptional expression. A total of 12 *PAL* genes were identified in the common walnut and clustered into two subfamilies based on phylogenetic analysis. These common walnut *PAL*s are distributed on eight different pseudo-chromosomes. Seven of the 12 *PAL*s (*JrPAL2-3*, *JrPAL4-2*, *JrPAL2-1*, *JrPAL4-1*, *JrPAL8*, *JrPAL9*, and *JrPAL6*) were specific found in *J. regia*, and *JrPAL3*, *JrPAL5*, *JrPAL1-2*, *JrPAL7*, and *JrPAL2-2* were found to be closely associated with the woody plant *Populus trichocarpa*. Additionally, the expression patterns of *JrPAL3*, *JrPAL7*, *JrPAL9*, and *JrPAL2-1* showed that they had high expression in female and male flowers. The miRNA ath-miR830-5p regulates two genes, *JrPAL5* and *JrPAL1*, such that they have low expression in the male and female flowers of the common walnut. Our research provides useful information for further research into the function of *PAL* genes in common walnut and *Juglans*.

## 1. Introduction

Phenylalanine ammonia-lyase (PAL, EC4.3.1.5), the first enzyme of the phenylpropanoid pathway, produces precursors to a variety of important secondary metabolites, such as phytoalexin, lignin, and phenolic compounds [1,2,3,4,5]. PAL, first reported in 1961 [6], potentially comprises protective compounds, such as flavonoids, furanocoumarin phytoalexins, and cell wall components [7]. The *PAL* gene is widely present in higher plants, and is also found in some fungi, yeasts, and very few bacteria, but not in animals [8,9]. *PAL* encoding genes are generally well studied and are commonly found as small gene families comprising one to five members [10,11,12,13,14]. For example, four *PAL* genes have been identified and functionally characterized in *Arabidopsis thaliana* [15,16], five in *Populus trichocarpa* [17], three in *Scutellaria baicalensis* [18], and three in *Coffeac anephora* [19]. However, some studies have reported that the *PAL* gene family has more than five members; for example, 13 *PAL* genes have been found in *Cucumis sativus* [20], 12 in *Citrullus lanatus* [20], 13 in *Cucumis melo* [21], and 16 in *Vitis vinifera* [22]. In *Pinus taeda*, five distinct *PAL* genes have been identified [12]. In *Salix babylonica*, *PAL1*, *PAL2*, *PAL3*, and *PAL4* genes are orthologous to the poplar genes [13]. In *A. thaliana*, *AtPAL1* and *AtPAL2* are highly expressed in roots and mature flowers and are barely detectable in leaf tissues [15,16], and in most woody plants cluster into two clusters [13,14,17,20]. Moreover, low-temperature stress enhances *PAL* activity [23]. *CaPAL1* acts as a positive regulator in the phenylpropanoid pathway [24]. *PAL* functions as a positive regulator of rice allelopathic potential [25].

Common walnut (*Juglans regia* L.), also known as English walnut, Persian walnut, or hú táo in Chinese, is an economically important hardwood tree species cultivated worldwide for its high quality and quantity of wood, nutritious nuts, and traditional medicinal value [26,27,28,29,30]. The *PAL* gene (JX069977.1) has been cloned in the common walnut as *PAL* [31] and its expression has been studied [32]. Transcription analysis revealed that *JrPAL* was expressed in all tested tissues including roots, stems, and leaves, with the highest transcription level found in roots [31]. From June to September, the related *JrPAL* expression was most pronounced in August, indicating a high synthesis rate of phenolic compounds at this development stage in different varieties of *J. regia* [33]. In black walnut (*J. nigra*), *PAL* genes were also strongly expressed in autumn, suggesting that their transcription in these tissues contributes to phenolic biosynthesis [34]. In a previous study, increases in total phenolics in harvested fresh walnut kernels due to cold stress were accompanied by increases in *PAL* specific and total activity [32]. These studies show that the *PAL* gene plays an important role in plant growth and explore its evolutionary mechanism and expression pattern, which lays a foundation for further research on gene function, which is a meaningful perspective for studying the evolutionary mechanism of the *PAL* gene. However, comprehensive information and functional characterization of the *PAL* gene family of the common walnut (*J. regia*) still remain unclear, especially the expression in male and female flowers.

In this study, we systematically characterized 12 *PAL* genes in the common walnut. We used an iterative process of manual and computational analysis to identify members of the common walnut *PAL*-encoding gene family within the latest released common walnut whole-genome sequence [29]. We constructed a phylogenetic tree of *PAL* genes. Conserved motifs, gene structure, protein domain, chromosome localization, and miRNA prediction were further analyzed in common walnut *PAL* genes. To understand the function of the *PAL* genes in the common walnut, we also studied the transcriptional-level expression profiles of *PAL*s in male and female flowers at different development stages. Our results provide useful theoretical support for the functional characterization of these *PAL* genes that are involved in the flower development process in common walnut.

## 2. Materials and Methods

### 2.1. Identification of *PAL* Genes in the Common Walnut (*Juglans Regia*)

To identify putative *PAL* gene family members in *J. regia*, we performed BLASTP (Protein-protein Basic Local Alignment Search Tool) searches in the common walnut genome database, version 1.0 (https://treegenesdb.org/FTP/Genomes/Jure/), with a top E value less than 1 × 10^−20^ [35]. The available PAL protein sequences were obtained from the following sources: the dicotyledons *A. thaliana* (At) and *P. trichocarpa* (Pt), and the monocotyledons *Zea mays* (Zm) and *Oryza sativa* (Os) from the Ensembl Plants database (http://archive.plants.ensembl.org/info/website/ftp/index.html) [35]. The walnut whole protein database was downloaded from the National Center for Biotechnology (NCBI) genome plant database (https://www.ncbi.nlm.nih.gov /genome/17683). All walnut PAL potential candidate protein sequences were examined using the Pfam database (http://pfam.xfam.org) [36], the Conserved Domain Database (CDD) (cdd/wrpsb.cgi), and the Simple Modular Architecture Research Tool (SMART) database (http://smart.embl-heidelberg.de/) with an E value cutoff of 1.0 by the domain analysis programs [37]. Sequences lacking PAL–TAL motifs were removed using ClustalX version 2.1 software with default parameters to verify potential common walnut PAL protein candidates by comparing all of the protein sequences with known PAL proteins [38].

### 2.2. Analysis of Protein Sequence Properties

The PAL sequence name and position information was acquired through BLAST with the parameters E-value < 10^−15^ and ID % > 50%. [39]. The PAL sequences were predicted on the Plant-mPLoc website to predict subcellular localization of plant proteins, including those with multiple sites [40]. The theoretical isoelectric point and molecular weight were predicted in the ProtParam tool (https://web.expasy.org/protparam/) [41].

### 2.3. Phylogenetic Analysis

Multiple sequence alignments were generated using ClustalX version 2.1 with default parameters [38]. The evolutionary relationship of *J. regia PAL* proteins with *A. thaliana*, *Z. mays*, *O. sativa*, and *P. trichocarpa* PAL proteins was studied using MEGA7 [42]. A phylogenetic tree was constructed using the neighbor-joining (NJ) and maximum likelihood (ML) methods using MEGA version 7.1, the tree topology support was assessed by means of a bootstrap analysis with 1000 replicates, and the phylogenetic tree displayed bootstrap values greater than 50 [42]. The syntenic relationship of *PAL* genes in the common walnut was conducted using Multiple Collinearity Scan Toolkit (MCScanX) [43]. Initially, potential gene pairs (E-value < 10^−5^, top 5 matches) obtained by BLASTP across *J. regia* genomes, were used as input file for MCScanX to analyze segmental and tandem duplications [35,43].

### 2.4. Conserved Domain and Motifs Displayed in *J. Regia* PAL Proteins

The motifs were identified using the Multiple EM for Motif Elicitation (MEME) program with default parameters (http://meme-suite.org/) [44]. The parameters were as follows: the maximum number of motifs was set to 20 and the optimum motif width was set to 30–50. We searched each motif sequence of the *PAL* genes using the SMART database (http://smart.embl-heidelberg.de/) with default parameters. The maximum number of motifs was set to 20, with conserved domains through the NCBI-Batch-CDD software [37].

### 2.5. Analysis of Gene Exon–Intron Structures

The exon–intron structure of each *J. regia PAL* gene was confirmed from alignment of the coding sequence (CDS) with the corresponding common walnut genomic sequences through the est2genome program (http://emboss.bioinformatics.nl/cgi-bin/emboss/est2genome). The exon–intron structure was illustrated using the online Gene Structure Display Server program (http://gsds.cbi.pku.edu.cn) by comparing their CDS with genomic sequences of *A. thaliana* (At) and *P. trichocarpa* (Pt) by aligning the FASTA-formatted CDS and genomic DNA sequences [1,45,46]. Related walnut gene sequences were searched for on the Genome Browser (https://www.ncbi.nlm.nih.gov/genome/).

### 2.7. Chromosome Location of Common Walnut *PAL* Genes

The chromosomal locations of *J. regia PAL* genes were generated using MapInspect software [47] (http://www.softsea.com/download/MapInspect.html) based on the initial position information. The required chromosomal location information was downloaded from the Ensembl Plants database (http://archive.plants.ensembl.org/Triticum_aestivum/Info/Index). To further explore the ways of gene replication, the CDS sequences of the *J. regia PAL* genes were compared homologously using ClustalX version 2.1 with default settings [38].

### 2.8. miRNA Predicted in Common Walnut *PAL* Family Genes

All of the genome sequences of the common walnut *PAL* family genes were submitted as candidate genes to predict potential miRNAs by searching against the available walnut reference of miRNA sequences using the psRNATarget Server with default parameters [48]. We visualized the interactions between the predicted miRNAs and the corresponding target walnut *PAL* genes using Cytoscape software with default parameters [49].

### 2.9. Plant Materials, Treatments, Collections, and RNA Isolation and Analysis of Gene Expression Profiles

To assess the expression of common walnut *PAL* genes, we collected 12 samples of fresh female and male flowers of individual common walnut trees grown in the Qinling Mountains, which were collected at different development stages (1 to 3 biological replications, on 10, 15, and 22 April, and 1 May), frozen in liquid nitrogen prior to storage at −80 °C until use (Table S1). Total RNA was isolated by an RNA-prep Pure Plant Kit (Tiangen, Beijing, China) [50]. Libraries for RNA sequencing (RNA-seq) were produced using a NEBNext Ultra RNA Library Prep Kit (NEB, Beverly, MA, USA). Paired-end sequencing was performed on the Illumina HiSeq2500 platform to generate 100 bp reads with default parameters by Novogene Bioinformatics Technology Co. Ltd., Beijing, China. The de novo transcriptome was assembled using default settings in Trinity based on the well genome reference of *J. regia* [29,51]. To further characterize the different temporal and spatial gene expression patterns of the *JrPAL* gene family, we analyzed RNA-seq data. The transcriptome sequencing datasets were deposited in BioProject under ID PRJNA358784, which was used to perform RNA-seq of different *J. regia* wild female and male flowers. We analyzed the total RNA-seq data of the female and male flowers at the initial germination flowering stages. We quantified these gene expression levels on the basis of their fragments per kilobase of transcript per million mapped reads (FPKM) values using Cufflinks with default parameters [52], and represented these results using HemI 1.0 software with default parameters [53]. The differential gene expression (DESeq) analysis was performed using the DESeq R package (1.10.1). Genes with an adjusted *p*-value < 0.05 found by DESeq were assigned as differentially expressed [54]. We normalized the number of reads for the differential gene expression from the RNA-seq data based on a method described by Anders and Huber using the DESeq Bioconductor package with default parameters [55,56,57]. Unigenes were annotated using data from the NCBI Gene Ontology (GO) and Pfam databases. GO annotations were performed in Blast2GO v2.5 with a cutoff E value of 1 × 10^−6^ [58].

## 3. Results

### 3.1. Identification and Characterization of Common Walnut *PAL* Genes

A total of 12 full-length genes coding putative phenylalanine ammonia-lyase (PAL) were identified in the *J. regia* genome (Table 1). The 12 sequences containing PAL-HAL domains belong to the *PAL* gene family. It is evident that these 12 sequences form a family, named the *PAL* gene family. These walnut *PAL* proteins ranged in length from 281 to 760 amino acids, with molecular weights from 31.19 kDa to 83.97 kDa and isoelectric points ranging from 5.58 to 8.75. Subcellular localization analysis indicated that all 12 walnut *PAL* genes are localized in the cytoplasm (Table 1).

### 3.2. Phylogenetic Relationship of Common Walnut and Other Four Plants in the *PAL* Gene Family

**Figure 1:**
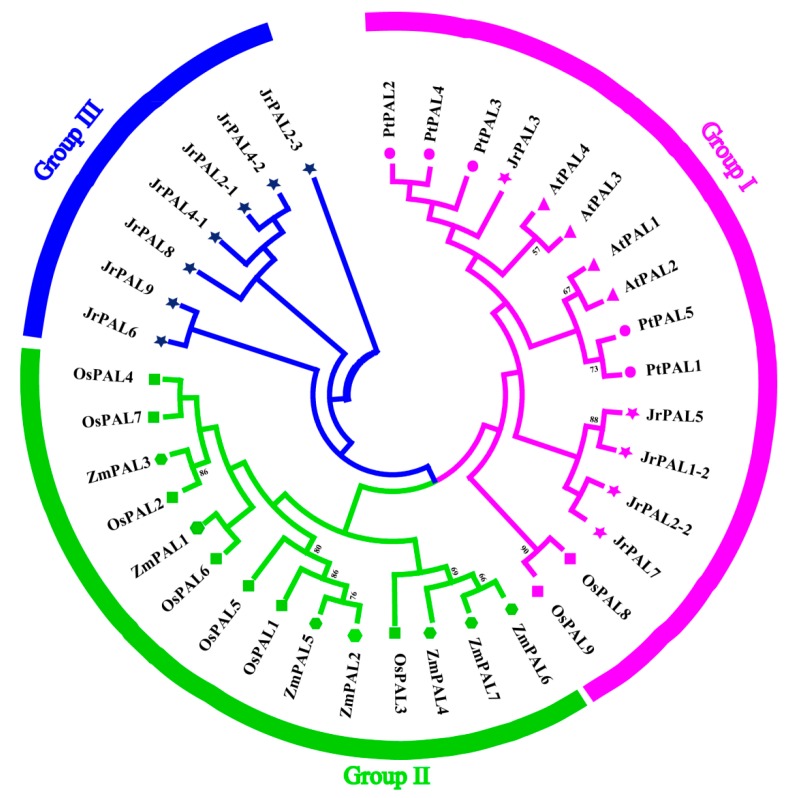
Phylogenetic analysis of phenylalanine ammonia-lyase (PAL) proteins among common walnut (12), *Arabidopsis* (4), rice (7), maize (6), and poplar (5). These 34 sequences were used to construct a neighbor-joining (NJ) tree. The tree was divided into three groups, represented by different colors. Triangles represent *Arabidopsis*, rectangles represent rice, pentagons represent maize, circles represent poplar, and pentagrams represent walnut. The number on each node of branch indicates the bootstrap support value more than 50.

To investigate the lineage-specific expansion of *PAL* genes in the *J. regia* genome, we performed a phylogenetic analysis of all *PAL*s from *J. regia*, *A. thaliana* proteins, *O. sativa* proteins, *Z. mays* proteins, and *P. trichocarpa* proteins. These plants had 12, 4, 7, 6, and 5 *PAL* genes, respectively. Based on the completed alignment of the sequences, *PAL*s were clustered into three groups, designated group I, group II, and group III (Figure 1). Overall, group I contained 7 *PAL*s, group II contained 14 *PAL*s, and group III contained 14 *PAL*s (Table S2). Seven *PAL* genes (*JrPAL2-3*, *JrPAL4-2*, *JrPAL2-1*, *JrPAL4-1*, *JrPAL8*, *JrPAL9*, and *JrPAL6*) were specifically found in *J. regia*, and five common walnut *PAL*s (*JrPAL3*, *JrPAL5*, *JrPAL1-2*, *JrPAL7*, and *JrPAL2-2*) were found to be closely associated with *P. trichocarpa* (Figure 1 and Figure S1).

### 3.3. Position, Conserved Motifs, and Exon–Intron of Common Walnut *PALs*

**Figure 2:**
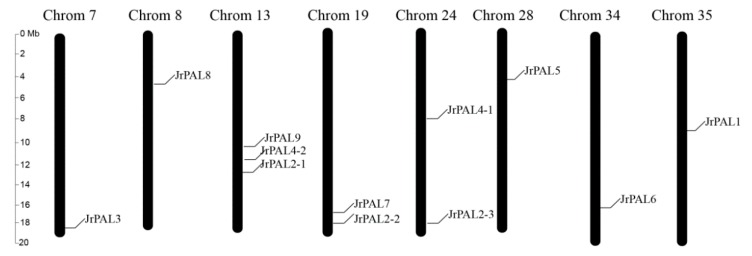
Chromosome locations of *PAL* genes of common walnut on 40 scaffolds (pseudo-chromosomes). The pseudo-chromosome name is at the top of each bar. The scale of the pseudo-chromosomes is millions of base pairs (Mb).

The 12 *PAL* genes were assigned to eight pseudo-chromosomes of *J. regia* based on the physical positions (Figure 2; Table 1). The distribution of the *PAL* genes was different on each pseudo-chromosome. Pseudo-chromosome 13 contained the largest number of *PAL* genes (3), followed by pseudo chromosome 19 (2 genes) and pseudo-chromosome 24 (2 genes). All other pseudo-chromosomes contained one gene (Figure 2; Table 1). *JrPAL3* and *JrPAL2-2* is pairs of segmentally duplicated *PAL*s in the *J. regia* genome. (Figure 2; Table 1).

**Figure 3:**
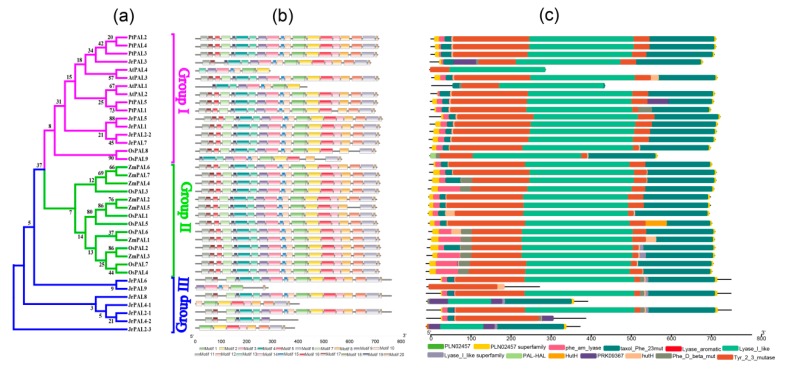
(**a**) Phylogenetic relationships, (**b**) motif compositions, and (**c**) conserved domains of the 12 *PAL* genes identified in the common walnut. Phylogenetic relationships used the neighbor-joining (NJ) method, and different colors represent different groups. Colored boxes indicate conserved motifs and gray lines represent nonconserved sequences. The lengths of motifs in each protein are shown proportionally.

The differences within the PAL family were further analyzed by examining the conserved motifs and domains using the MEME program and the NCBI-CDD database, respectively. After going through the MEME program, the result of the *PAL* genes was 20 motifs. The predicted walnut *PAL* gene motifs ranged from 8 to 50 amino acids in length. Motif 1 was mostly present in all species except *JrPAL4-2* (Figure 3a,b). Common walnut *PAL*s containing the 13 domains belong to the *PAL* family, and PRK09367 domains exist in group I and group III; in group II it is a specific domain, and the Lyase_I_like domain is a conserved domain in the three groups. These results suggest that all *PAL* genes in the walnut contain at least one typical domain (Figure 3b,c).

**Figure 4:**
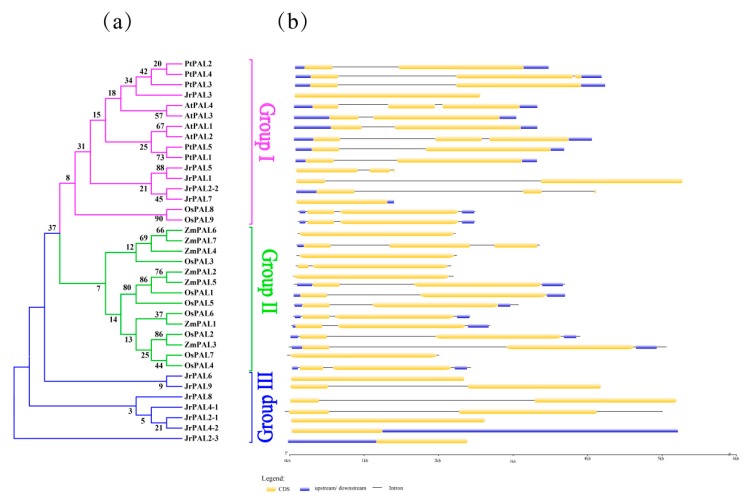
(**a**) Phylogenetic relationships and (**b**) gene structures of *PAL* genes in *Arabidopsis*, poplar, rice, maize, and common walnut. CDS, upstream/downstream, introns, and intron insertion are shown. Orange boxes indicate coding sequence, blue boxes indicate upstreams or downstreams genes, and gray lines represent introns.

The number of introns per gene varied from one to two based on the exon–intron organization structure analysis of *PAL* genes in *Arabidopsis*, common walnut, rice, maize and poplar (Figure 4). The intron positions of orthologous *PAL* genes in *Arabidopsis* and poplar and their insertion with symmetric exons are well conserved, indicating that all these *PAL* genes might have a common ancestor. The structure of *PAL* genes of common walnut have a different exon–intron organization compared with *Arabidopsis* and poplar (Figure 4). The exon–intron organization structure of *A. thaliana* and *P. trichocarpa* shows an intron insertion in the front, and none exists in *J. regia* (Figure 4). The gene structure of the *PAL* gene family in *J. regia* has an intron in the middle (Figure 4). Overall, the whole gene length of woody plants is longer than that of herbs (Figure 4b).

### 3.4. MicroRNA Targeting and Expression Profile Analysis of Common Walnut *PAL* Genes

**Figure 5:**
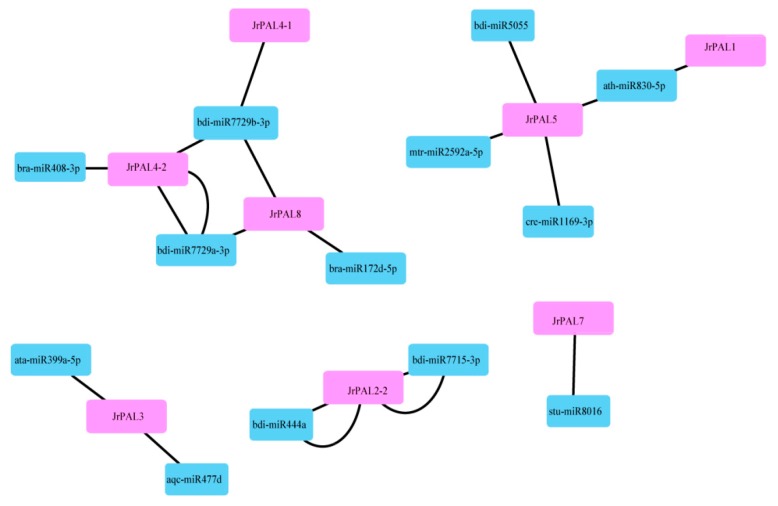
A schematic representation of the regulatory network relationships between the putative miRNAs and their targeted walnut *PAL* genes.

**Figure 6:**
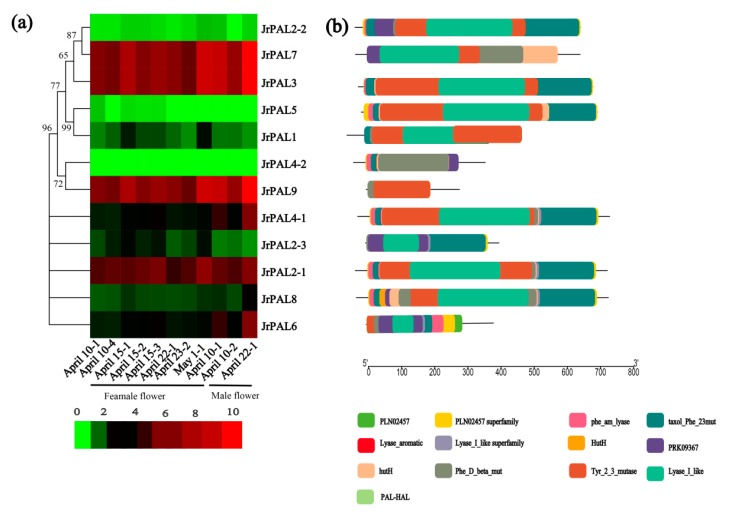
(**a**) Phylogenetic relationships and expression patterns and (**b**) conserved motif compositions of the 12 *PAL* genes in walnut. The phylogenetic tree was constructed based on full-length protein sequences using MEGA6.0 (http://web.megasoftware.net/) with hierarchical clustering of the relative expression levels of *PAL* genes. RNA-seq data of female and male flowers in common walnut were used to analyze expression patterns. The heat map was drawn in log10-transformed expression values. Red and green represent decreased and increased expression levels in each sample, respectively; 1 represents one biological replication; 2 represents two biological replications; 3 represents three biological replications; 4 represents four replications. The Multiple EM for Motif Elicitation (MEME) program was used to predict conserved motifs. Each motif is represented by a different colored box. Heat map shows expression patterns of walnut *PAL* family genes in six stages. Red and green represent relatively high and low expression compared to control, respectively.

To understand the underlying regulatory mechanism of miRNAs involved in the regulation of *PAL*s, we identified 13 putative miRNAs targeting 12 common walnut *PAL* genes to construct a relationship network using Cytoscape software (Figure 5; Table S3). We analyzed the connection distribution of the regulation network and found that *JrPAL4-2* is one of the most targeted *PAL* genes of common walnut. The bdi-miRNA 7729b-3p targets walnut genes *JrPAL*4-1, *JrPAL4-2*, and *JrPAL*8, *JrPAL*5, *JrPAL7*, *JrPAL2-2*, *JrPAL3* (Figure 5). Our results show that the miRNA ath-miR830-5p targets *JrPAL5* and *JrPAL1*. The two genes *JrPAL5* and *JrPAL1* have low expression in common walnut flowers (Figure 6). Furthermore, our results also show that the miRNA bdi-miR7729b-3p targeting *JrPAL4-1* has high expression in flowers; while three miRNAs (bdi-miR7729b-3p, bdi-miR408-3p, and bdi-miR7729a-3p) targeting *JrPAL4-2* have high expression in common walnut flowers (Figure 5 and Figure 6).

To gain insight into the putative functions of walnut *PAL* genes, the temporal and spatial expression profiles of the identified *PAL* genes were examined using the RNA-seq data (Figure 5; Table S4). Four *PALs* (*JrPAL7*, *JrPAL3*, *JrPAL9*, and *JrPAL2-1*) had high expression in female and male flowers (Figure 5). We found that the *PAL* genes with high expression had almost a Tyr_2_3 mutase motif. The expression results showed that the maturity of male flowers had increased expression in all *PAL* genes (Figure 5). In addition, the phylogenetic relationships are close, and the expression patterns of the two genes are quite different, resulting in *JrPAL7*, *JrPAL3*, *JrPAL9*, and *JrPAL2-1* missing motif PRK09367.

## 4. Discussion

### 4.1. Characteristics of Phenylalanine Ammonia-Lyase Gene in *J. Regia*

Phenylalanine ammonia-lyase (PAL) is the first enzyme in the phenylpropanoid pathway [1,27]. PAL plays a critical role in plant growth, development, and adaptation. Recently, there have been reports on the functional analysis of *PAL* genes in the common walnut [1,27,31,32]. In this study, we found that *JrPAL2-2* has a high similarity with *JrPAL* (JX069977.1) [31] (Table S5). At the molecular level, the identification and isolation of the *PAL* gene are very important, as the gene was shown to be closely related to stress resistance in previous studies [1,23,24]. Increases in specific and total activity of *PAL* during cold storage is consistent with the previous study, suggesting that the *PAL* gene family in the walnut may also have a basic function of resisting stress from cold, salt, and disease [32]. Recently, some studies have shown that the *PAL* gene is localized to the cytoplasm [13,59,60]. Our results also show that all 12 *PAL* genes were predicted to be in the cytoplasm of the common walnut (Table 1).

### 4.2. Comparisons of *PAL* Gene Family in Plants; Expansion and Evolution of Common Walnut *PALs*

The *PAL* gene family members have significant differences among various plant species (Figure 1). We identified 12 *PAL* genes (*JrPAL1-12*) in *J. regia* based on the complete reference common walnut genome sequence (Table 1; Figure 1). Our results show that the number of *PAL* genes in *J. regia* far exceeds the four *AtPALs* in modern *A. thaliana*, suggesting that genome duplication may have occurred in the evolution of *J. regia* [15,16]. From *JrPAL1* to *JrPAL12*, despite differences in the genome size and the total number of protein-encoding genes among the sequenced plant species, the number of *PAL* genes seemed not to increase or decrease proportionally (Table 1) [20]. We searched the *PAL* genes in 40 common walnut scaffolds. (pseudo-chromosomes) by anchoring the scaffolds to the walnut linkage groups (Table S6) [61]. These 12 *PAL* genes were localized to eight scaffolds in the common walnut (Table 1; Figure 2) [61]. Duplication events are important in the expansion and evolution of gene families, including whole-genome duplications, small-scale segmental duplications, local tandem duplications, or combinations of these possibilities [62,63,64]. The one gene pair (*JrPAL3* and *JrPAL2-2*) choose the segmental duplicated events in common walnut, which might be caused by gene expansion based on their chromosomal distribution and phylogenetic and syntenic relationships (Figure 2) [20,43].

Based on phylogenetic analysis, our 12 *JrPAL*s were separated into two distinct groups, as *PAL* genes from cucurbit species *A. thaliana* [15,16,20], suggesting similar evolutionary trajectories between *J. regia* and cucurbit species. The phylogenetic tree constructed for *PAL* genes was divided into four clusters in *C. lanatus* [65], four clusters in *P. taeda* [12], and two clusters in most woody plants (*S. babylonica*, *Ornithogalum saundersiae*, and *P. trichocarpa*) [13,14,17] (Figure 1). Motif 1 was only detected in walnut *PAL* protein sequences compared to other plants (Figure 1 and Figure 3b), indicating that the walnut *PAL* gene family has a highly conserved protein structure. The *PAL* genes of these five plants contain all the conserved domains identified in NCBI-Batch-CDD, suggesting that the *PAL* gene family is extremely conservative in evolution, presuming that *PAL* genes have important antiretroviral effects. The key domain is phe_am_lyase, which exists in all genes including all species. In group I, *JrPAL3*, *JrPAL5*, *JrPAL1*, *JrPAL2-2*, and *JrPAL7* are distributed in different subclades of the phylogenetic tree in Figure 3c, mainly because of specific PRK09367 domains in the front of the protein structure. *JrPAL2-1* and *JrPAL4-2*, and *JrPAL6* and *JrPAL9* show quite a difference in protein structure length, but they exist on the same branch of the phylogenetic tree, and we can notice that Tyr_2_3_mutase domains are distributed in the protein structure. These genes (*JrPAL2-1*, *JrPAL4-2*, *JrPAL6*, and *JrPAL9*) compared to the other genes (*JrPAL4-1* and *JrPAL2-3*) have a clear difference in protein structure, which is the lack of the obvious major domain PRK09367. A phylogenetic analysis of walnut *PAL* genes shows that they share similar motifs in each subfamily (Figure 3a,b), while motif 1 may be consistent with Tyr_2_3_mutase, so it can be deduced that motif 1 will perform the function of Tyr_2_3_mutase (Figure 3c).

Studying exon–intron gene structure can provide important clues for gene evolution [65]. Genome-wide characterization, molecular cloning, and expression analysis of the structure of *PAL* genes in walnut and the overall gene structure of *Arabidopsis* show great differences in exon and intron regions, and one of them showed the same performance (Figure 4a,c) [1]. Compared to the structure of the *CiPAL* gene in watermelon, the structure of *PAL* in common walnut has undergone evolutionary changes [66]. There is genetic similarity in *A. thaliana* and *C. lanatus* and *PAL* gene families [65,66]. The information of *PAL* gene structure in walnut and poplar indicates that the *PAL* family has undergone major variations in evolution [66] (Figure 4c).

### 4.3. Comprehensive Analysis of microRNAs Targeting Common Walnut *PAL* Genes and Expression Levels of *PALs* in Common Walnut Flowers

In recent years, many studies have shown that miRNAs in plants mainly respond to stress by regulating the expression of genes associated with stress [67]. *JrPAL* is expressed in roots, shoots, and leaves, but little is known about its expression in flowers [31]. There is no doubt that ath-miR830-5p plays an important role in *A. thaliana* by targeting two genes with lower expression in flowers than in other tissues; for example, roots and leaves [67,68]. Our experiments of RNA-seq from female and male flowers and miRNA prediction of *PAL* genes in the common walnut indicated that ath-miR830-5p targeting *JrPAL5* and *JrPAL1* has low expression in common walnut flowers (Figure 5).

As an entry point to the phenylpropanoid pathway, *PAL* is tightly regulated at the pre- and post-transcriptional levels. In previous studies, the *PAL* genes showed different expression patterns in different organs (xylem, roots, leaves, stems, and flowers) in willow, *C. sativus*, and *Salvia miltiorrhiza* [11,13,15,16,17,18]. However, there have been few reports on the *PAL* gene family in common walnut flowers. *AtPAL1* and *AtPAL2* were shown to be highly expressed in *Arabidopsis* flowers, while *JrPAL3* also showed high expression in female and male flowers of the common walnut (Figure 6) [15]. Furthermore, we found that *AtPAL1* and *AtPAL2* were clustered into the same group with *JrPAL3* based on the phylogenetic tree (Figure 1 and Figure 6). Therefore, it is speculated that the *PAL* gene is more important at the time of male flower maturation, which can be explained from the side, as the plant becomes more resistant as a mature individual than a young individual (Figure 6b). Different members of gene families generally exhibit disparities in abundance in different tissues or with different stressors [69,70]. This correlation indicates that *PAL*s with similar evolutionary status might play a similar role in plant growth, which allowed us to investigate the functions of *PAL*s from other cucurbits using a comparative genomic approach. The results showed that six genes (*JrPAL7*, *JrPAL3*, *JrPAL9*, *JrPAL4-1*, *JrPAL2-1*, and *JrPAL6*) have high expression in both female and male flowers; another six genes (*JrPAL2-2*, *JrPAL5*, *JrPAL1*, *JrPAL4-2*, *JrPAL2-3*, and *JrPAL8*) have a low expression pattern in both female and male flower tissues. This result of *PAL* gene family members is due to the different protein structure, gene structure, and microRNA network between the former six genes (high expression) and the latter six genes (low expression) ( Figure 3, Figure 4 and Figure 6). We found that the expression patterns of the *PAL* gene family in various growing stages of female and male flowers are similar, which shows that they are resistant genes in plant flowering. *JrPAL2-2*, *JrPAL7*, *JrPAL3*, *JrPAL5*, *JrPAL1*, *JrPAL4-2*, *JrPAL2-1*, *JrPAL8*, and *JrPAL9* have the same expression level at different stages of female and male flowers, but *JrPAL6* and *JrPAL4-1* have high expression in male flowers compared to female flowers (Figure 6a). Changes in expression levels during the female and male flowering process indicated the important role of *PAL* genes in sex determination and resistance adaptation. In addition, the expression of *JrPAL1* and *JrPAL3* was upregulated at the May 1 time point for female flowers, and the expression of *JrPAL8* was upregulated at the 22 April time point for male flowers, indicating their different roles during the flowering process. The diverse expression patterns of the *PAL* family genes indicate a complex regulation of the *PAL*-mediated phenylpropanoid pathway during the flowering process of the common walnut. The structure of domain Lyase_I_like of *JrPAL2-3* was different between female and male flowers. Meanwhile, *JrPAL2-3* has high expression in female flowers compared to male flowers, which indicates that the gene domain might affect the pattern of expression of the plant-flowering process (Figure 6). In addition, the microRNAs bra-miR408-3p, bdi-miR7729b-3p, and bdi-miR7729a-3p target the gene *JrPAL4-2*, and bdi-miR7729b-3p, bra-miR172d-5p, and bdi-miR7729a-3p target the gene *JrPAL8* (Figure 5). Moreover, the microRNA ath-miR830-5p targets two genes, *JrPAL5* and *JrPAL1*; however, they have low expression levels in both female and male flowers at the whole growth stage (Figure 5 and Figure 6). With the same pattern, the microRNAs bdi-miR7715-3p and bdi-miR444a both target gene *JrPAL2-2*, which has low expression in female and male flowers. The two genes *JrPAL7* and *JrPAL3* have high expression levels among the *PAL* gene family in the common walnut; *JrPAL 7* is targeted by the microRNA stu-miR8016, and *JrPAL3* is targeted by two microRNAs, ata-miR399a-5p and aqc-miR477d. The diverse patterns of microRNA-targeted *PAL* genes indicate that the networks of microRNA may be key regulator network for the *PAL* gene family in the common walnut.

## 5. Conclusions

We identified 12 *PAL* genes in the common walnut. These members are distributed on eight pseudo-chromosomes. The *PAL* genes were divided into two subfamilies. Seven *PAL*s (*JrPAL2-3*, *JrPAL4-2*, *JrPAL2-1*, *JrPAL4-1*, *JrPAL8*, *JrPAL9*, and *JrPAL6*) were specifically found in *J. regia*, and *JrPAL3*, *JrPAL5*, *JrPAL1-2*, *JrPAL7*, and *JrPAL2-2* were found to be closely associated with *P. trichocarpa*. The exon–intron gene structure of *PAL*s in the common walnut indicated that the *PAL* family has undergone major variations in evolution compared with *Arabidopsis*. The relative expression levels of PALs varied in different developmental stages of female and male flowers of common walnut; *JrPAL3*, *JrPAL7*, *JrPAL9*, and *JrPAL2-1* were expressed at high levels in most samples. The transcriptional level of *JrPAL6* increased in the flowers with the expression of all *PAL*s at different developmental stages. The miRNA ath-miR830-5p targeting *JrPAL5* and *JrPAL1* has a low expression in common walnut flowers. These findings could lay a theoretical foundation for the functional study of *PAL*s and the further construction of common walnut light regulation networks.

## Figures and Tables

**Table 1:** Phenylalanine ammnia-lyase (PAL) gene family information in common walnut (*J. regia*).

Gene Name	Protein ID	Gene ID	CDS ID	Subcellular Location	Amino Acids (aa)	Molecular Weight (kDa)	Theoretical pI	Chromosome	Chromosome Length	Gene Position	
										Start	End
*JrPAL3*	XP_018828772.1	NW_017443600.1:c859630-855244	XM_018973227.1	Cytoplasm	708	77.04	5.96	Chr7	19,001,705	18,431,863	1,8427,477
*JrPAL5*	XP_018859391.1	NW_017437924.1:10925-15347	XM_019003846.1	Cytoplasm	712	77.71	6.06	Chr28	18,296,634	2,940,954	2,945,376
*JrPAL2-2*	XP_018844813.1	NW_017389549.1:c62915-57410	XM_018989268.1	Cytoplasm	680	74.11	5.91	Chr19	18,508,379	1,8341,787	18,337,338
*JrPAL2-1*	XP_018827035.1	NW_017443587.1:471936-474695	XM_018971490.1	Cytoplasm	760	83.67	6.16	Chr13	18,490,500	1,2030,270	12,033,029
*JrPAL4-1*	XP_018845411.1	NW_017389589.1:c87043-84575	XM_018989866.1	Cytoplasm	760	83.92	6.38	Chr24	18,356,466	3,643,363	3,640,895
*JrPAL8*	XP_018817312.1	NW_017443031.1:261176-263621	XM_018961767.1	Cytoplasm	760	83.97	6.35	Chr8	19,363,960	5,898,473	5,900,918
*JrPAL1*	XP_018853318.1	NW_017394290.1:c1301-6	XM_018997773.1	Cytoplasm	432	47.05	6.57	Chr35	18,286,198	9,845,144	9,843,849
*JrPAL2-3*	XP_018845408.1	NW_017389589.1:c6632-4075	XM_018989863.1	Cytoplasm	402	44.94	5.74	Chr24	18,356,466	3,562,298	3,560,395
*JrPAL4-2*	XP_018827000.1	NW_017443587.1:336894-338297	XM_018971455.1	Cytoplasm	397	44.11	8.75	Chr13	18,490,500	11,895,228	11,896,204
*JrPAL6*	XP_018855337.1	NW_017419648.1:c5073-3779	XM_018989863.1	Cytoplasm	384	42.92	5.85	Chr34	18,288,579	16,505,050	16,503,756
*JrPAL7*	AAX18624.1	NW_017389549.1:c62915-57410	XM_018989268.1	Cytoplasm	289	31.19	5.58	Chr19	18,508,379	18,341,787	18,337,338
*JrPAL9*	XP_018827002.1	NW_017443587.1:394996-399270	XM_018971457.1	Cytoplasm	281	31.47	6.40	Chr13	18,490,500	11,954,327	11,956,842

Note: Protein ID, Gene ID, and CDS (coding sequence) ID indicate that the accession number of the *PAL* gene family member sequences were downloaded from the National Center for Biotechnology (NCBI). kDa indicates that kilodaltons (unified atomic mass unit), pI indicates that isoelectric point.

## Supplementary Materials

The following are available online at http://www.mdpi.com/2073-4425/10/1/46/s1, Figure S1. Phylogenetic analysis of PAL proteins among common walnut (12), *Arabidopsis* (4), rice (7), maize (6), and poplar (5). These 34 sequences were used to construct a maximum likelihood (ML) tree. Table S1. A total of 12 samples of female and male flowers of common walnut were used for expression profiling in this study. Table S2. Distribution of common walnut *PAL* genes in Class I, II, and III. Table S3. The putative miRNAs and their targeted *JrPAL* genes. Table S4. Expression data of *PAL* genes in flowers at different developmental stages in common walnut (*J. regia*). Table S5. Kinship between JX069977.1 and *JrPAL*1-12 in common walnut. Table S6. Information on 40 pseudo-chromosomes for common walnut (*J. regia*).
