# Supplementary figures and images for: Genome-Wide Identification and Transcriptional Expression of the PAL Gene Family in Common Walnut (Juglans Regia L.)
Source: Genes (Basel). 2019 Jan 15;10(1):46. doi: 10.3390/genes10010046 (PMC6357058; doi:10.3390/genes10010046)

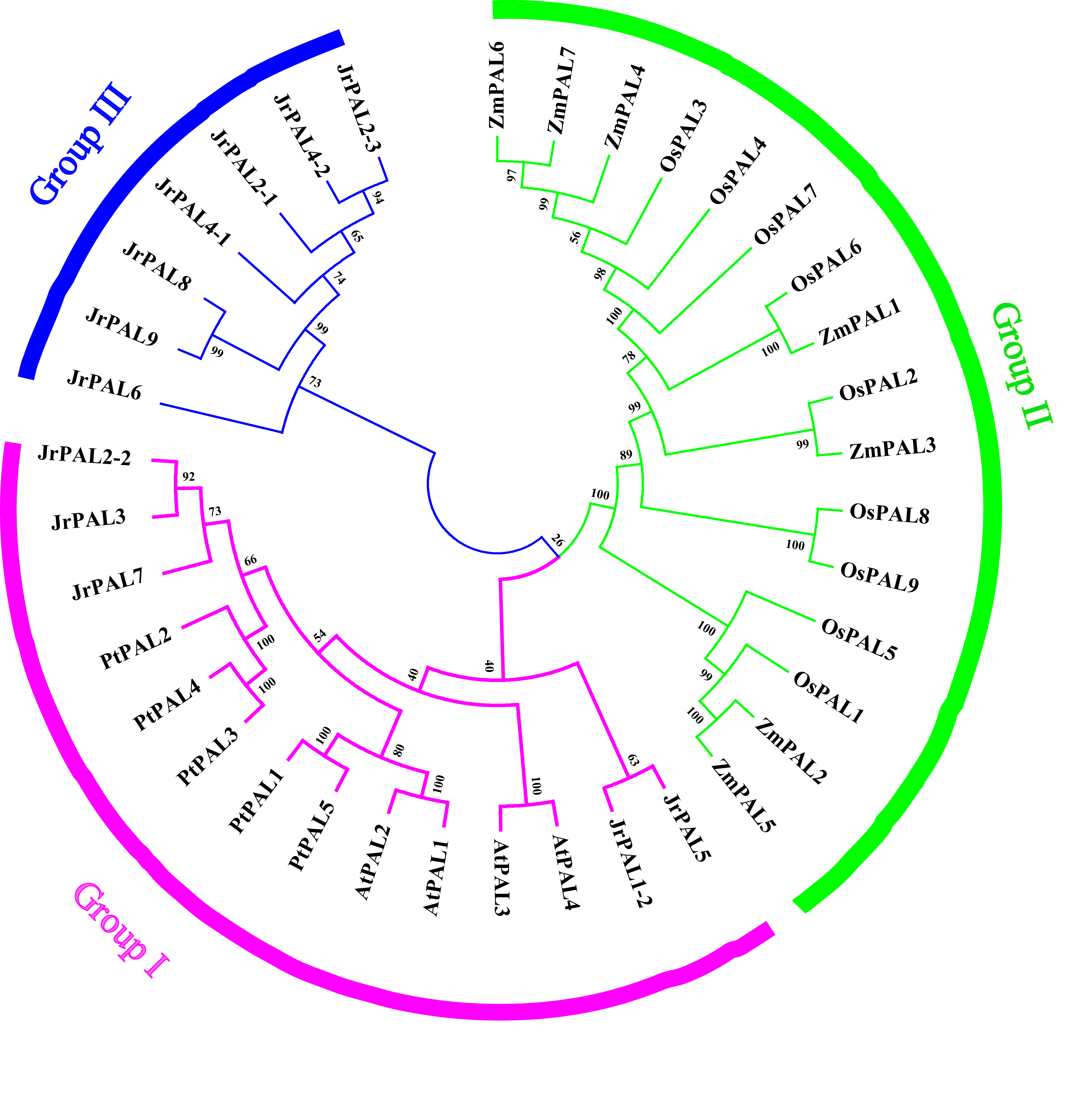

Supplement: Supplementary file 1 [file genes-10-00046-s001.zip › Supplementary materials/Figure S1.tif]
